# 361. Think Fungus! – Clinical profile, Risk factors and Diagnostic Utility of Galactomannan in diagnosis of Invasive Aspergillosis in Non-Neutropenic patients – A Prospective Study from India

**DOI:** 10.1093/ofid/ofac492.439

**Published:** 2022-12-15

**Authors:** Akshatha Ravindra, Deepak Kumar, Gopal Krishna Bohra, T R Neetha, Yash Khatod, Durga Shankar Meena, Naresh Kumar Midha, Vidhi Jain, Kuldeep Singh, Nishant Kumar Chauhan, Ankur Sharma

**Affiliations:** AIIMS Jodhpur, Jodhpur, Rajasthan, India; AIIMS jodhpur, Jodhpur, Rajasthan, India; AIIMS, Jodhpur, Rajasthan, India; AIIMS Jodhpur, Jodhpur, Rajasthan, India; AIIMS Jodhpur, Jodhpur, Rajasthan, India; AIIMS, Jodhpur, Rajasthan, India; AIIMS, Jodhpur, Rajasthan, India; AIIMS, Jodhpur, Rajasthan, India; AIIMS Jodhpur, Jodhpur, Rajasthan, India; AIIMS Jodhpur, Jodhpur, Rajasthan, India; AIIMS Jodhpur, Jodhpur, Rajasthan, India

## Abstract

**Background:**

Invasive aspergillosis(IA) is known to occur in immunocompromised patients including neutropenic patients. But there has been a trend of increasing cases in non-neutropenic host with the emergence of newer risk factors like DM, cirrhosis etc.

The aim of this study was to evaluate the clinical features & risk factors of IA in non-neutropenic patients & to look at the clinical utility of galactomannan in diagnosis of IA.

**Methods:**

This was a prospective observational study which included the suspected cases of IA, based on the clinical & radiological criteria. Patients with haematological & solid organ malignancy were excluded. In patients with suspected Invasive pulmonary aspergillosis (IPA), serum & BAL, while in patients with suspected CNS IA CSF & serum samples were sent for galactomannan analysis (Platelia ELISA). The clinical features, risk factors, outcomes were analysed.

**Results:**

We screened 243 patients with suspected IA, of which 49 non-neutropenic patients with IA (16 Proven & 33 Probable cases) were included. The mean age was 47.8 years. Of all IA cases 69.5% (n=34) were IPA, 20.4% (n=10) were CNS aspergillosis & 10.2% (n=5) showed disseminated form of IA. The common symptoms included Fever (71.4%), cough (71.3%), expectoration (44.7%) & dyspnoea (59.1%) in IPA, while in CNS aspergillosis, presented with fever (73.3%), altered sensorium (53%).The predominant risk factor included previous TB, DM, COVID-19. The radiological manifestations in IPA included the typical cavity (40.4%, n=17), Centrilobular nodules with tree in bud appearance in 56.5% (n=23). The CNS aspergillosis was associated with ring enhancing lesion (41.6%, n=5) with leptomeningeal enhancement (50%, n=6), while cerebral abscess was seen in 16.6% (n=2) patients. The positivity of galactomannan were 24.4%, 91.3% & 87.5% in serum, BALF & CSF respectively. Culture positivity & Direct smear positivity was 18.3% & 28.5% respectively. The overall mortality was 20.4%. Complete response in 3 months follow-up period was seen in 69.3% patients.

**Conclusion:**

The clinical manifestations of IA in non-neutropenic are diverse & nonspecific. Also, culture & direct microscopy lack sensitivity, hence diagnostic markers like Galactomannan can be used for early diagnosis of IA in patients with newer emerging risk factors.

**Disclosures:**

**All Authors**: No reported disclosures.

